# Mixed autoimmune hemolytic anemia as the initial presentation of systemic lupus erythematosus: A case report and review

**DOI:** 10.1002/jha2.1008

**Published:** 2024-09-11

**Authors:** Brian P. Edwards, Sidhartha Gautam Senapati, Mariia Kasianchyk, Joel Shah, Fatih Ayvali, Satish Maharaj

**Affiliations:** ^1^ Department of Internal Medicine Texas Tech University Health Sciences Center El Paso Texas USA; ^2^ Department of Oncological Sciences Moffitt Cancer Center University of South Florida Tampa Florida USA

**Keywords:** mixed‐type autoimmune hemolytic anemia, positive antiphospholipid antibodies, severe autoimmune hemolytic anemia, systemic lupus erythematosus

## Abstract

Autoimmune hemolytic anemia (AIHA) is an acquired condition caused by autoantibody mediated destruction of erythrocytes. AIHA is classified as warm or cold depending on whether the autoantibodies involved react optimally at or below body temperature (37°C), respectively. Mixed AIHA, with features of both, is rare and clinically more severe. We report a case of mixed AIHA that was found to be the presentation of systemic lupus erythematosus (SLE). Treatment with rituximab and prednisone resulted in good response. Although more commonly associated with warm AIHA, SLE can present with mixed AIHA.

## 1 INTRODUCTION

Autoimmune hemolytic anemia (AIHA) is an acquired condition from autoantibody‐mediated destruction of red blood cells (RBC) with incidence estimated at 1–3 cases per 100,000 per year [1, 2, 3, 4, 5]. The clinical spectrum of AIHA ranges from mild to severe disease with estimated mortality up to 10% [5]. Serologically, AIHA may be warm (65%), cold (30%), or mixed (5%) [1]. Warm AIHA is caused by immunoglobulin G (IgG) that opsonizes RBCs at body temperature, while cold AIHA is caused by immunoglobulin M (IgM) that binds to RBC at temperatures below body temperature leading to complement fixation and primarily extravascular hemolysis [1, 2] In mixed AIHA, there is the presence of both warm and cold autoantibodies.

AIHA may manifest as idiopathic or primary in 50% of cases, or secondary to various conditions such as lymphoproliferative syndrome (20%), autoimmune diseases (20%), infections, and tumors [1, 2]. Regarding autoimmune conditions, specifically systemic lupus erythematosus (SLE), AIHA has been reported in up to 10% of patients [3, 4]. AIHA in SLE is typically associated with warm autoantibodies, and rarely is mediated by cold autoantibodies [3, 4].

### 1.1 Case report

A 22‐year‐old Hispanic female presented with generalized weakness, fatigue, and intermittent body aches for 1 month. Symptoms worsened over the 3 days prior and were accompanied by nausea, headache, dizziness, chest pain, and dyspnea. Vital signs on admission showed that the patient was afebrile, tachycardic (124 beats/min), tachypneic (18 breaths/min) and BP 121/60 mm Hg, and 99% oxygen saturation on room air. Physical exam findings revealed a young female in mild distress, with icterus, conjunctival pallor, and jaundice.

**FIGURE 1:**
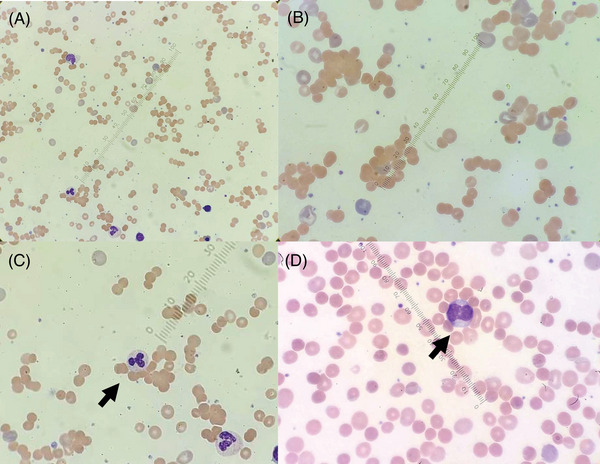
(A,B) Peripheral blood smear, demonstrating erythrocyte agglutination with abundant spherocytes and reticulocytes. (C,D) Leukocyte–erythrocytes rosettes (arrows) are seen, a finding strongly associated with clinical AIHA. These are thought to form from surface Fc receptors interacting with IgG‐bound erythrocytes.

Laboratory findings on admission are presented in Table 1; showing severe hemolytic anemia (hemoglobin 4.9 g/dL), elevated lactate dehydrogenase with undetectable haptoglobin, reticulocytosis, and hyperbilirubinemia. Platelet and leukocyte counts were preserved, with no coagulopathy and slightly elevated D‐dimer, normal serum B12 level and no glucose‐6‐phosphate dehydrogenase deficiency. Peripheral blood smear showed widespread erythrocyte agglutination with abundant spherocytes and reticulocytes (Figure 1A,B). Furthermore, leukocyte–erythrocytes rosettes were noted (Figure 1C,D); a finding strongly associated with clinical AIHA.

**TABLE 1:** Laboratory testing on admission.

Test	Results	Reference range
***Peripheral blood***		
White blood cell (/µL)	9.85 × 10^3^	4.50–11.00 × 10^3^
Hemoglobin (g/dL)	4.9	12.0–15.0
Hematocrit (%)	14.7	36.0 –47.0
Mean corpuscular volume (MCV, fl)	116.7	82.0–98.0
Reticulocyte count (%)	13.3	0.5–1.5
Platelets (/µL)	226 × 10^3^	150–450 × 10^3^
***Blood chemistry***		
Total bilirubin (mg/dL)	4.3	0.2–1.3 mg
Direct bilirubin (mg/dL)	0.70	0.00–0.30
Lactate dehydrogenase (LDH, IU/L)	904	120–246
Haptoglobin (mg/dL)	<8	43–212
Vitamin B12 (pg/mL)	429	239–931
Glucose‐6‐phosphate dehydrogenase (U/g)	>21.0	7.0–20.5

Direct antiglobulin testing (DAT) was positive for both complement C3 and IgG. A cold antibody screen was positive, and the cold agglutinin titer was positive (> 1:64). Serum immunofixation and electrophoresis were performed but did not show a monoclonal component. Testing for viral infection, cryoglobulins and biphasic hemolysin (Donath Landsteiner test) were negative. Therefore, with both warm and cold reacting autoantibodies and RBC agglutination on review of the peripheral blood, mixed AIHA was diagnosed. Serological studies for autoimmune disease (Table 2) revealed high titer antinuclear antibodies, anti‐Smith antibodies, decreased complement C3/C4, positive lupus anticoagulant and antiphospholipid antibodies (APS). Based on consensus criteria, the diagnosis of mixed AIHA as presentation of SLE was made.

**TABLE 2:** Autoimmune serology.

Lab	Results	Reference range
ANA	Positive	–
ANA titer	1:2560	<1:160
Anti‐dsDNA	Negative	
Anti‐Sm	>8.0	<1.0
Anti‐Ro (SSA)	<1.0	<1.0
Anti‐La (SSB)	<1.0	<1.0
Anti‐U1‐RNP	>8.0	<1.0
Complement (CH_50_), total (U/mL)	23	31–60
C3 (mg/dL)	55	88–165
C4 (mg/dL)	<8.0	14.0–44.0
Lupus anticoagulant	Positive	–
β2‐glycoprotein‐I antibodies (β2GPI) (IgG, U/mL)	>112.0	<20.0
β2‐glycoprotein‐I antibodies (β2GPI) (IgA, U/mL)	20.2	<20.0
β2‐glycoprotein‐I antibodies (β2GPI) (IgM, U/mL)	88.5	<20.0
Phosphatidylserine/prothrombin antibody (IgG, U)	16	≤30
Phosphatidylserine/prothrombin antibody (IgM, U)	>150	≤30
Cardiolipin antibody (IgG, U/mL)	71.3	<20.0
Cardiolipin antibody (IgM, U/mL)	70.7	<20.0
Cardiolipin antibody (IgA, U/mL)	22.7	< 20.0

**FIGURE 2:**
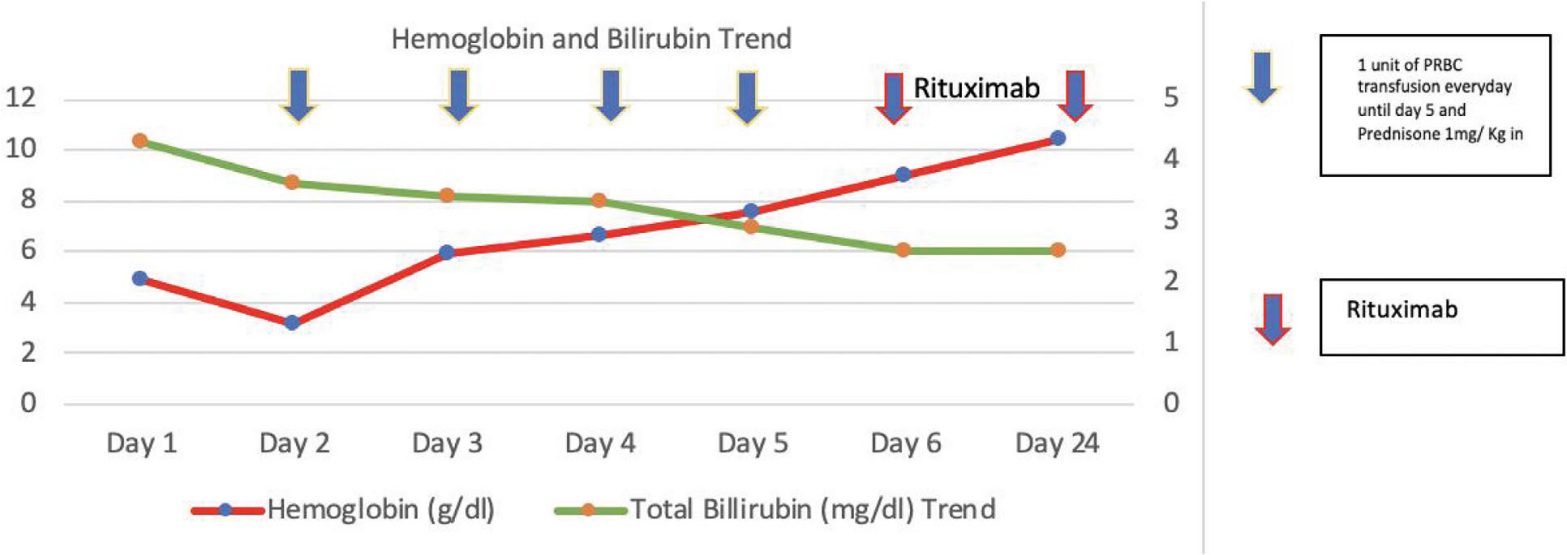
Trend in hemoglobin and bilirubin with response to treatment.

Serial testing showed worsening anemia (hemoglobin 3.2 g/dL). Prednisone 1 mg/kg was initiated, and one unit of packed RBC was transfused slowly daily until the fourth day of hospitalization. On day 6 of hospitalization, rituximab 375 mg/m^2^ intravenous infusion weekly was commenced; the patient was discharged with hemoglobin 8.4 g/dL the day after. Outpatient follow up continued, for a total of four doses rituximab, and slow prednisone taper. Two weeks after completing the rituximab course, hemoglobin was 11.1 g/dL and the patient was clinically improved (Figure 2).

## 2 DISCUSSION

AIHA is caused by increased RBC destruction triggered by autoantibodies reacting against RBC antigens, with or without complement activation [1, 2, 5, 6]. It is a clinicopathologic diagnosis characterized by hemolysis and positive autoantibody testing. In warm AIHA, IgG autoantibodies react with erythrocytes at 37°C [1, 2]. RBC bound IgG is a weak activator of the classical complement pathway [2] but activates the monocyte–macrophage system via Fcϒ interactions; leading to extravascular phagocytosis of opsonized RBCs, primarily in the spleen and to a lesser extent liver [2, 5]. Leukocyte–neutrophil rosettes (Figure 1C,D) have been proposed to represent a physiologic intermediate in extravascular RBC destruction and strongly predict clinical AIHA.

In cold AIHA, primarily IgM autoantibodies react optimally below 37°C [2], leading to agglutination and complement activation via the classical pathway. C3b‐coated erythrocytes are phagocytosed by macrophages in the reticuloendothelial system, mainly in the liver. Activation via the classical pathway can also produce complement split products leading to the formation of a membrane attack complex and intravascular hemolysis [3, 7]. Mixed AIHA, caused by a combination of a warm IgG and cold IgM autoantibody, presents with hemolytic anemia that is more severe and chronic [1, 5, 7].

SLE is an autoimmune inflammatory disease of unclear etiology [1, 4], primarily affecting women of childbearing age and more common in Hispanic and African‐Americans, than in Caucasians [4, 8]. Hematologic manifestations of SLE include most commonly anemia, but also leukopenia and thrombocytopenia [4]. The etiology of anemia may be multifactorial, including anemia of chronic disease, autoimmune hemolysis, renal disease, or treatment‐induced [4]. Autoimmune hemolysis occurs in up to 10% of SLE patients [1, 3, 4]. Hemolytic anemia may manifest years before or after the diagnosis of SLE, although it is seldom the initial presenting feature [4]. Warm autoimmune hemolytic anemia (AIHA) is the type most commonly seen in SLE [3, 4].

Management of AIHA depends on classification. In warm AIHA, corticosteroids remain first line, using prednisone at 100 mg fixed dose or 1–1.5 mg/kg daily; slowly tapering after 2–3 weeks and discontinuing at 4–6 months [5, 7]. If secondary, treating the underlying condition is recommended if steroids prove ineffective [7]. For cold AIHA, steroids are often ineffective, as is splenectomy given extravascular hemolysis primarily occurs in the liver [5, 7]. Instead, therapies that target the pathogenic B‐cell clone such as rituximab, or the classical complement pathway, such as sutimlimab, have efficacy [5, 7, 9]. Rituximab at the dose of 375 mg/m^2^/week for 4 weeks is the most widely used first‐line therapy [7].

In mixed AIHA, the disease may respond to steroids, and patients may require multiple lines of therapy before responding [1, 7]. There is a low rate of long sustained remission after tapering and discontinuation of steroids, particularly in severely affected patients. The consequence is that patients may become steroid dependent or managed on very high doses. Two prospective, randomized trials investigated the addition of rituximab upfront to patients with warm AIHA.

One rituximab regimen consisted of 4 infusions of 375 mg/m^2^ weekly and the other 2 infusions of 1000 mg fixed dosing, 2 weeks apart [7, 10]. Both studies had similar results, showing twice the rate of long‐term responses in patients who had been given the combination as compared to those treated with prednisolone alone [7, 10]. As a result, the first international consensus group has recommended that severely affected patients, which include those patients with Hb < 8 g/dL, patients with Evans’ syndrome, and patients with atypical AIHA which includes IgA‐mediated, DAT‐negative and mixed, should be considered for rituximab plus prednisolone combination therapy in the first line [7, 11]. In this case of mixed AIHA with underlying SLE, the first line combination regimen was efficacious and well‐tolerated.

## 3 CONCLUSION

This case reminds clinicians that while mixed AIHA as an initial presentation of SLE is rare, it can present in a life‐threatening manner. In evaluating mixed AIHA, serology for SLE should be considered. The combination therapy consisting of steroids and rituximab can induce durable responses and is recommended.

## AUTHOR CONTRIBUTIONS

**Brian P. Edwards**: Design; conduct; manuscript drafting. **Sidhartha Gautam Senapati**: Design; conduct; manuscript drafting. **Mariia Kasianchyk**: Design; conduct; manuscript drafting. **Joel Shah**: Pathological analysis; manuscript drafting. **Fatih Ayvali**: Design; conduct; manuscript drafting. **Satish Maharaj**: Design; conduct; manuscript drafting.

## CONFLICT OF INTEREST STATEMENT

The authors declare no conflicts of interest.

## FUNDING INFORMATION

The authors report no sources of funding apply to this report.

## ETHICS STATEMENT

Data collected in accordance with institutional protocols and patient consent obtained and available on request.

## PATIENT CONSENT STATEMENT

The participant has consented to the submission of the case report to the journal.

## CLINICAL TRIAL REGISTRATION

The authors have confirmed clinical trial registration is not needed for this submission.

## Data Availability

Further data may be available from the corresponding author upon reasonable request.
